# Pulmonary Artery Banding for Ventricular Rehabilitation in Infants With Dilated Cardiomyopathy: Early Results in a Single-Center Experience

**DOI:** 10.3389/fped.2020.00347

**Published:** 2020-07-16

**Authors:** Angela Di Candia, Biagio Castaldi, Giulia Bordin, Alessia Cerutti, Elena Reffo, Roberta Biffanti, Giovanni Di Salvo, Vladimiro L. Vida, Massimo A. Padalino

**Affiliations:** ^1^Pediatric Cardiology Unit, Department of Woman and Child's Health, University of Padua, Padua, Italy; ^2^Pediatric and Congenital Cardiac Surgery Unit, Department of Cardiac, Thoracic and Vascular Sciences and Public Health, University of Padua, Padua, Italy

**Keywords:** clinical management, end-stage heart failure, infants, surgery, outcomes

## Abstract

**Background:** Pulmonary artery banding (PAB) is reported as an innovative strategy for children with end-stage heart failure (ESHF) to bridge to transplantation or recovery. We report our early experience with PAB to evaluate outcomes, indications, and limitations.

**Materials and Methods:** This is a single-center prospective clinical study, including infants and children admitted for ESHF owing to dilated cardiomyopathy (DCM) with preserved right ventricular function after failure of maximal conventional therapy. All patients underwent perioperative anticongestive medical therapy with ACE inhibitor, beta blocker, and spironolactone. Post-operatively, all patients underwent echocardiographic follow-up to assess myocardial recovery.

**Results:** We selected five patients (four males) who underwent PAB at a median age of 8.6 months (range 3.9–42.2 months), with preoperative ejection fraction (EF) <30%. Sternal closure was delayed in all. One patient did not improve after PAB and underwent Berlin Heart implantation after 33 days, followed by heart transplant after 13 months. Four patients were discharged home on full anticongestive therapy. However, 2 months after discharge, one patient experienced severe acute heart failure secondary to pneumonia, which required mechanical circulatory support, and the patient underwent a successful heart transplant after 21 days. The remaining three patients are doing well at home, 22.4, 16.9, and 15.4 months after PAB. They all underwent elective percutaneous de-banding, 18.5, 4.8, and 10.7 months after PAB. EF increased from 17.7 ± 8.5% to 63.3 ± 7.6% (*p* = 0.03), and they have all been delisted.

**Conclusion:** Use of PAB may be an effective alternative to mechanical support in selected infants for bridging to transplant or recovery. Better results seem to occur in patients aged <12 months. Further experience and research are required to identify responders and non-responders to this approach.

## Introduction

Significant advances in end-stage heart failure (ESHF) therapy have been achieved and documented in adult patients in recent years, whereas the mechanisms and therapy of heart failure in children are still unknown (1, 2). The ultimate therapy for ESHF is heart transplantation (HT) (3, 4). However, this is not readily available in infants and children (5–7). Therefore, novel therapeutic strategies are needed.

Long-term mechanical circulatory support (MCS) is currently possible and effective even in neonates and infants with ESHF, thanks to the extracorporeal Berlin Heart EXCOR®, which is the only currently available ventricular assist device (VAD) in patients weighing <10 kg (5, 8). However, the incidence of major lethal or disabling complications is not negligible, and the management of Berlin Heart often requires long-term hospitalization with increased hospital costs and patient discomfort (5).

On the basis of the hypothesis that ventricle–ventricular interaction can benefit dilated left ventricular failure, Schranz et al. (1) proposed an innovative application of the pulmonary artery banding (PAB) as an effective treatment of ESHF in dilated cardiomyopathy (DCM) in young children (<6 years of age) with preserved right ventricular function. A multicenter experience with PAB for DCM in selected pediatric patients has been recently reported, (9) with satisfactory results. However, no clear indications have been described, and open debate is still ongoing whether this procedure is valid, or these patients would experience recovery anyway from viral myocarditis.

On the basis of this background and experiences, since 2015, we have embarked on the “PAB strategy” for treating ESHF in infants. We herein report the early results of our ongoing clinical experience, aiming at outlining indications, outcomes, and limitations.

## Materials and Methods

This is a single-center, prospective, clinical study including infants and children with DCM since September 2015. Inclusion criteria were as follows: age <4 years, evidence of left ventricle dysfunction, and preserved right ventricle function; being listed for transplant, as indicated elsewhere (1); and failure of conventional inotropic therapy, with more than two intensive care unit (ICU) admissions within the same hospitalization. Excluded were patients with biventricular failure, moderate tricuspid regurgitation, idiopathic or reactive pulmonary hypertension, and associated major congenital heart disease (CHD; such as the anomalous origin of a coronary artery from the pulmonary artery). Preoperative, intraoperative, and post-operative data were retrieved from our institutional database, including clinical and follow-up periodical reports. The patients' echocardiographic evaluation was always done by two cardiologists (AC and BC). The local hospital committee on clinical investigation approved the review of medical records, and individual patients' data were anonymized. Parental consent for salvage procedure was obtained.

Before PAB, all patients underwent full congestive heart failure (CHF) medical therapy, as recommended by Schranz et al. (2) (lisinopril, bisoprolol, and spironolactone). Whenever clinical condition was unstable, the patient was admitted in pediatric ICU and intubated and mechanically ventilated, and intravenous infusion of inotropes (dopamine and milrinone) and levosimendan were started. It is of note that as suggested by Schranz (2), furosemide was used only in case of extreme oliguria (<1 cm^3^/kg/h).

Echocardiographic evaluation was focused on the estimation of left ventricular ejection fraction (LVEF) (calculated by the Simpson method), mitral and tricuspid valves regurgitation [according to European Society of Cardiology (ESC) recommendations], (10) and trans-PAB pressure gradient (assessed by continuous Doppler velocity gradient –dPmax = 4 × Vmax^2^). The right ventricle function was assessed using tricuspid annular peak systolic excursion (TAPSE). All these data were evaluated before and after PAB and at the last available follow-up evaluation.

All patients underwent PAB through a midline sternotomy, as described elsewhere (1), with a PTFE band, under continuous trans-esophageal echocardiography (TEE) monitoring. In particular, the PAB was tightened with 7.0 prolene stitches to facilitate later balloon dilation. Pulmonary artery pressure and right ventricle pressure were measured continuously through trans-thoracic intracardiac lines. The PAB was tightened to obtain a right ventricle pressure equal to 70% of systemic blood pressure, or until TEE was showing a leftward shift of the interventricular septum, or TAPSE reduction, or increasing tricuspid regurgitation. Chest closure was delayed in all patients to facilitate ventricular and pulmonary compliance recovery and to facilitate additional post-operative tightening of the PAB, if required.

### Statistical Analysis

All the analyses were performed using a commercially available package (SPSS, Rel 18.0 2009, SPSS Inc., Chicago). Quantitative values are presented as mean + 1 *SD* and median value. Continuous variables were compared by using a *t*-test for paired data. The null hypothesis was rejected for a *p* < 0.05.

## Results

Five patients (M/F = 4/1) with a median age of 8.6 months (range 3.9–42.2) underwent PAB for ESHF. Three patients were reporting symptoms like fever, malaise, and upper respiratory tract infection before admission. All patients were negative at autoimmune and inborn metabolic disease screening. Three patients were found to be positive for viral infection (Table 1). Pathological analysis of trans-parietal right and left ventricle myocardial tissues showed active lymphocytic myocarditis in four and chronic myocarditis in one. Besides, endocardial fibroelastosis was found in two.

**Table 1:** Anamnestic, histological, and microbiologic preoperative data.

**Patient#**	**Gender**	**Age (m)**	**Weight** **(kg, percentile)**	**Type of DCM**	**Histology**	**Aspecific symptoms prior admission**	**Viral test**
1	M	8.6	8.0, 10th percentile	LV non-compaction cardiomyopathy [heterozygous mutation of TPM1 gene° (*de novo*) and of ABCC9 gene (parental)]	Not performed	Yes	Not performed
2	M	42.2	12.0, 3rd percentile	Fulminant myocarditis in idiopathic unrecognized DCM	Active lymphocytic myocarditis with endocardial fibroelastosis	Yes	Negative
3	M	3.9	5.9, 10th−25th percentile	Acute myocarditis	Active lymphocytic myocarditis with endocardial fibroelastosis	No	CMV-DNA positive on blood and urine samples, negative in myocardial biopsy
4	F	12.5	7.7, <3rd percentile	PVB19 chronic myocarditis	Chronic myocarditis in activity Phase	Yes	PVB19 on blood sample and myocardial biopsy
5	M	5.7	4.8, <3rd percentile	Acute myocarditis	Active lymphocytic myocarditis	No	HHV6-DNA positive on blood sample, genomic integration in patient's cells, negative in myocardial biopsy

CMV, cytomegalovirus; DCM, dilated cardiomyopathy; PVB19, parvovirus B19; LV, left ventricular.

*°associated with non-compaction cardiomyopathy*.

On admission, the mean baseline EF was 15.4 ± 6.8%; mitral valve regurgitation was moderate in four and mild in one patient; mean TAPSE value was 11.1 ± 4.1 mm. Last, tricuspid regurgitation was trivial or mild in all. No patient required extracorporeal membrane oxygenation (ECMO) support before PAB (Table 2).

**Table 2:** Preoperative echocardiographic findings.

**Patient#**	**Ross class**	**Preoperative MV**	**Initial BNP/p-BNP**	**LVEF**	**TAPSE**	**LVEDD**	**LVEDV**	**TR**	**MR**
				**(%)**	**(cm)**	**(cm/m^**2**^)**	**(ml/m^**2**^)**		
1	IV	Yes	10,226^*^	11	0.9	–	188	1	Moderate
2	IV	Yes	3,565^*^	13	1.5	–	–	1	Moderate
3	IV	Yes	3,892^*^	9	0.85	10.96	128	1	Mild
4	IV	Yes	58,059^§^	26	0.7	11.30	204	2	Moderate
5	III	No	5,031^§^	18	1.61	16.23	176	1	Moderate

BNP, brain natriuretic peptide; p-BNP, pro-BNP; LVEDD, left ventricular end-diastolic diameter; LVEDV, left ventricular end-diastolic volume; LVEF, left ventricular ejection fraction; MR, mitral valve regurgitation; MV, mechanical ventilation; TAPSE, tricuspid annular peak systolic excursion; TR, tricuspid valve regurgitation.

* BNP.

§ *p-BNP*.

**Figure 1:**
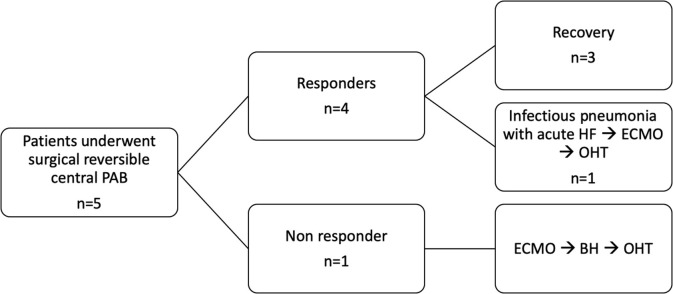
Synopsis. PAB, pulmonary artery banding; FU, follow-up; ECMO, extracorporeal membrane oxygenation; BH, Berlin Heart; OHT, orthotopic heart transplant.

There were no intraoperative deaths (Table 3, Figure 1). After PAB, TEE monitoring clearly showed decreased mitral valve regurgitation and a leftward shift of interventricular septum in all. All patients underwent a delayed sternal closure after a median time of 3 days (range 2–9 days). The PAB was tightened in four over five patients in ICU, and the median trans-PAB pressure gradient at transthoracic echocardiogram (TTE) at chest closure was 30 mmHg (range 30–55 mmHg).

**Table 3:** Intraoperative and post-operative data.

**Patient#**	**HT listed**	**Associated procedure (days)**	**MV (days)**	**Complications**	**Delayed sternal closure (days)**	**ICU stay**	**Hospital stay (days)**
1	Yes	None	8	None	2	20	39
2^*^	Yes	Atrial septostomy + ECMO	–^°^	LCO^*^	9	–^°^	–^°^
3	Yes	None	12	None	3	20	54
4	Yes	None	6	None	2	10	36
5	Yes	None	12	None	5	15	93

ECMO, extracorporeal membrane oxygenation; HT, heart transplant; ICU, intensive care unit; LCO, low cardiac output syndrome; MV, mechanical ventilation; PAB, pulmonary artery banding; OHT, orthotopic heart transplant.

* owing to hemodynamic instability and left atrium dilation with left bronchus obstruction, the patient underwent atrial septostomy right after PAB and required ECMO for 7 days; owing to hemodynamic instability, the patient required implantation of Berlin Heart 33 days after PAB, and subsequent OHT 13 months after PAB.

° *he was weaned off from mechanical ventilation for the first time 14 days after Berlin Heart implantation*.

The perioperative course was complicated in one patient only (#2), a 3.5-year-old male who was admitted emergently, already intubated, with left atrial massive dilation, which was compressing the left mainstem bronchus. Unlike the other patients, this child underwent PAB and decompressive atrial septostomy on pump. Furthermore, owing to unstable hemodynamic conditions, ECMO support was required for 7 days. Despite weaning off ECMO, his hemodynamic condition remained unstable, with EF <30%, requiring massive inotropic support and mechanical ventilation, Thus, he underwent elective left Berlin Heart EXCOR® implantation 33 days after PAB.

When the last patient was excluded, the median ventilation time, ICU, and hospital stay were 9.5 (6–12), 16.3 (10–20), and 39 days (35–54), respectively. All remaining patients were discharged home with full CHF therapy (lisinopril, bisoprolol, and spironolactone).

At a median follow-up of 30.2 months (25.4–55.7), all patients are alive and well. Two patients required HT after PAB. Patient #1 experienced acute pneumonia 3 months after PAB and required hospitalization in another hospital, where he had severe low cardiac output syndrome and required emergent venous arterial (VA) ECMO support. He was listed for transplant, and he underwent Berlin Heart EXCOR implantation. After 7 days of VAD support, he was successfully heart transplanted. He is currently in excellent conditions 48 months after transplantation, with no residual disabilities.

Patient #2, as mentioned above, underwent a successful HT after 12 months of uneventful VAD support, is in excellent clinical conditions 40 months after transplantation, and is on immunosuppressant therapy, with healthy neuropsychological development.

The remaining three patients had a progressive improvement of symptoms and left ventricular function. Follow-up data are presented in Table 4. Two of them (patients #3 and #4) had a complete cardiac function recovery. However, their early post-discharge period was characterized by relapsing signs of CHF, concomitantly to lung infections, which required rehospitalization and inotropic therapy with levosimendan 3 and 2.5 months after PAB, respectively. Patient #5 improved his cardiac function significantly but had no complete recovery yet, despite an uneventful clinical course. All of them underwent an elective percutaneous PAB balloon dilation 18.5, 4.8, and 10.7 months after PAB, respectively, because of the detection of progressive right ventricle hypertension and hypertrophy, with increased tricuspid regurgitation at control echocardiography. All patients tolerated this procedure well and had a substantial benefit from partial de-banding.

**Table 4:** Follow-up data.

**Patient#**	**Age at D/C (months)**	**FU time (months)**	**LVEF at 1 month (%)**	**LVEF at 3 months (%)**	**LVEF at 6 months (%)**	**LVEF at 12 months (%)**	**BD (days post-PAB)**	**BW** **(kg, percentile)**	**Medications**	**Ross class**	**Final outcome**
1	9.9	55.7	25	NA	NA	NA	NA	8, 10th percentile	Immunosuppressant	I	ECMO → HT^∧^
2*	55	49.2	13	NA	NA	NA	NA	12, 3rd percentile	Immunosuppressant	I	BH → HT^°^
3	5.7	37.5	24	19	30	56	566	13, 50th percentile	ASA, metoprolol, lisinopril	I	Recovery
4	13.7	30.2	32	27	22	19	149 and 233	11, 3rd−10th percentile	ASA lisinopril, bisoprolol, spironolactone, furosemide	I	Recovery
5	8.8	25.4	13	31	38	55	327	11, 10th−25th percentile	ASA, lisinopril, bisoprolol, spironolactone	I	Recovery

ASA, acetylsalicylic acid; BD, balloon dilation; BH, Berlin Heart; BW, body weight; D/C, discharge; ECMO, extracorporeal membrane oxygenation; FU, follow-up; HT, heart transplant; LVEF, left ventricular ejection fraction; NA, not applicable; PAB, pulmonary artery banding; OHT, orthotopic heart transplant.

∧ the patient experienced infectious pneumonia at home 2 months after PAB (another hospital), followed by cardiogenic shock; he was transferred to other hospital, where ECMO, and successful OHT were performed.

° *owing to hemodynamic instability, the patient required implantation of Berlin Heart 33 days after PAB and subsequent successful OHT 13 months after PAB. He was weaned off from mechanical ventilation for the first time 14 days after Berlin Heart implantation*.

**Figure 2:**
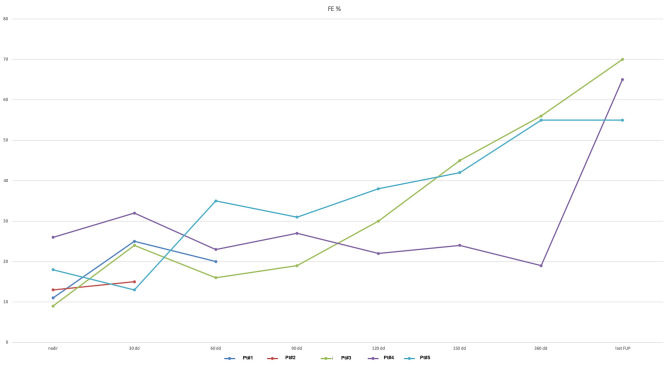
Left ventricular ejection fraction (EF) curve (expressed as %) shows improvement after PAB.

**Figure 3:**
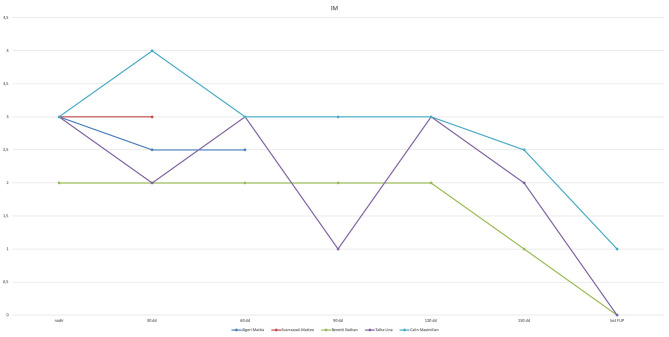
Mitral regurgitation (MR) degree curve shows improvement after PAB.

At the most recent follow-up (22.4, 16.9, and 15.4 months after PAB), these three patients are in Ross class I and thriving well. At the last echocardiography monitoring (Table 5), left ventricular end-diastolic diameter (LVEDD) decreased from a mean value of 12.8 ± 2.9 mm at baseline (*z*-score 11.4 ± 2.1) to 7.6 ± 1.8 mm (*z*-score 1.97 ± 3.14, *p* = 0.01), whereas EF increased to 63.3 ± 7.6% (*p* = 0.03 compared with baseline, Figure 2). Also, there was no or trivial mitral regurgitation (Figure 3), trivial or mild tricuspid regurgitation with preserved TAPSE values (*p* = 0.27 compared with baseline) and trans-pulmonary gradient of 50–55 mmHg. All patients had a gradual decrease in plasma levels of B-type natriuretic peptide (BNP)/pro-BNP, and they have all been ultimately removed from the transplant list, respectively 9, 10, and 12 months after PAB.

**Table 5:** Echocardiographic findings show improvement after PAB procedure in patients ^#^3, 4, and 5.

**Patient**	**^#^3**		**^#^4**		**^#^5**		**Mean** **±*****SD***		***p*-value**
**Echo parameters**	**Admission**	**Last FU**	**Admission**	**Last FU**	**Admission**	**Last FU**	**Admission**	**Last FU**	
Age (months)	3.9	22.4	12.5	16.9	5.7	15.4	–	–	–
LVEF (%)	9	70	26	65	18	55	17 ± 8.5	63.3 ± 7.6	0.03
LVEDD (cm)	10.9	5.3	11.3	5.8	16.2	8.5	12.8 ± 2.9	7.6 ± 1.8	–
LVEDD *z*-score	9.0	0	11.9	0.3	13.2	5.6	11.4 ± 2.1	1.9 ± 3.14	0.01
MR^^#^^	2	0	3	0	3	1	–	–	–
TR^^#^^	1	1	2	2	1	2	–	–	–
TAPSE (mm)	8.5	15.6	7.0	12.0	16	15.0	11.1 ± 4.1	14.2 ± 1.9	0.27

Note the significant improvement of LVEF and LVEDD z score.

FU, follow-up; LVEF, left ventricular ejection fraction; LVEDD, left ventricular end-diastolic diameter; MR, mitral valve regurgitation; TAPSE, tricuspid annular peak systolic excursion; SD, standard deviation; TR, tricuspid valve regurgitation.

# *0 = none; 1 = trivial; 2 = mild; 3 = moderate; 4 = severe*.

## Discussion

### Epidemiology and State of the Art of End-Stage Heart Failure Treatment in Pediatric Age

Population-based studies in the United States, Finland, and Australia estimate the incidence of primary cardiomyopathies to be 1/100,000 person-years in children <20 years of age (11, 12). Idiopathic DCM is the most common type in infants and children (13–15), and nearly 40% of children with symptoms, despite medical therapy, can develop progressive heart failure that may need lead to orthotopic heart transplant (OHT) or death within the first 2 years after diagnosis (11). Transplant-free survival in patients at 1 and 5 years is reported to be ~70 and 60%, respectively (13, 14).

Management of ESHF in infants and children is a challenging problem. Children with ESHF who cannot be stabilized with medical therapy can be supported effectively with MCS to unload the failing ventricle and maintain end-organ perfusion, (16, 17) as bridge to transplant or bridge to recovery.

Although some innovative centrifugal intrathoracic pumps have been successfully used in teenagers, (18) owing to dimensional restrictions, options for infants and smaller children remain limited to external devices, such as ECMO for short-term support, and extracorporeal VAD for long-term support, such as the Berlin Heart EXCOR®. Despite improved results through the years, invalidating VAD-related complications are still not infrequent (8). There is still an increased risk of infections and thromboembolic and bleeding events, which can potentially result in permanent neurological dysfunction or death, (16, 19) and local infection of the cables or the cannula insertion sites, which are extremely painful for the children and frustrating for caregivers. Furthermore, despite improved survival of children with ESHF, VAD utilization continues to have a prolonged ICU and hospital stay, waiting for a transplant or rarely recovery, which deteriorates the quality of life of patients and their families.

Despite the improvement in the medical management of ESHF patients, with the increased use of VAD and decreased frequency of rejection during the first year post-transplant, life after transplant is not easy, with a survival rate of about 90% 1 year after transplant and with overall 25-year survival of 37%, (20) which makes cardiac transplantation an unappealing option for a child. Last but not least, it is well-known that the incidence of neoproliferative diseases such as lymphoma or leukemia remains high in children after transplant, (21) limiting the effectiveness of HT in pediatric age.

### Pulmonary Artery Banding for Myocardial Rehabilitation

PAB is an old surgical technique described a long time ago as a palliative surgical procedure for CHD with pulmonary overflow. Despite the fact that early repair is currently the gold standard, PAB remains a valid option to balance pulmonary and systemic circulation in several complex CHD (9).

Based on previous experience derived from re-training of the subpulmonary left ventricle before anatomical correction in congenitally corrected transposition of the great arteries (TGA) or before arterial switch for dextro-TGA (D-TGA), (22–25) PAB has been applied to treat ESHF in children with DCM and preserved right ventricle function (26).

The first report of such application of PAB was published by the Giessen group in 2007 (27). The effectiveness of such a treatment in 12 patients was reported later by Schranz et al. (1, 28) as well as other groups over the past years (29, 30). A recent multicenter study has confirmed these promising outcomes in 70 patients worldwide (9).

The mechanism underlying the improvement of left ventricle function by right ventricle pressure overload induced by PAB is still uncertain. Most researchers believe that PAB causes a leftward shift of the interventricular septum that can reduce left ventricle dimensions, change its shape from spherical to ellipsoid morphology, and finally reduce the mitral valve annular dilatation and consequently mitral regurgitation, with the result of optimizing volume overload. Additionally, the PAB induces a favorable change in the Frank–Starling curve, and the reduction in left ventricle preload and end-diastolic/end-systolic filling pressures may enable a more favorable hemodynamic state and improvement of LVEF. Third, the PAB may determine an increase in right ventricle contractility (Anrep effect) and can stimulate its hypertrophy, matrix remodeling, and regenerative capacity of a young heart (31). Last but not least, PAB may enhance biological crosstalk between the right and left ventricles (i.e., the change in biventricular gene expression) that causes left ventricle reverse remodeling (co-hypertrophy) by favoring endogenous repair potential and can ultimately restore electromechanical right–left ventricle synchrony.

Some of these post-PAB effects have been partially described in a retrospective study based on cardiac MRI analysis presurgery and post-surgery of 15 children with DCM. This paper (31) showed that PAB leads to recovery of left ventricle size and global pump function, accompanied by improved left ventricle systolic strain, diastolic function, and intraventricular and interventricular synchrony. The right ventricle response to PAB consisted of a rise in mass with an increase in strain, associated with a leftward mechanical shift of the interventricular septum.

Improved left ventricle echocardiographic function and dimensions after PAB have been reported even in the setting of an experimental model of doxorubicin-induced left ventricle DCM (32). Despite all these exciting findings and data, we need further experimental work to elucidate the underlying molecular mechanism of this innovative strategy.

### Our Experience

Since 2015, we have applied the Giessen methodology to treat ESHF in selected infants and children. Our initial experience with PAB for ESHF has resulted in being an effective strategy to avoid or postpone HT in infants with DCM and preserved right ventricular function. Our population, despite small in number, is comparable in terms of age with that reported by the experience of other centers worldwide. We also report comparable data in terms of in-hospital and ICU stay, and mechanical ventilation time after surgery (9).

In four out of five patients, PAB showed to have an immediate effect in supporting left ventricle function, by the acute increase in right ventricle pressure. Mechanical remodeling, such as ventricular septum shift to the left, with a change in the shape of the left ventricle and a decrease in its preload and mitral valve regurgitation, was observed immediately after PAB. Besides, we found a significant and dramatic increase of LVEF and a decrease of LVEDD and blood level of BNP/pro-BNP at follow-up in those three patients who did not require a transplant.

It is of note that those patients, who initially responded positively to PAB (4/5 patients), presented a very different and complex clinical post-operative course. Two patients (1 and 4) experienced acute heart failure during pneumonia. Whereas patient #1 (DCM in non-compaction cardiomyopathy), owing to distance from our center, was urgently admitted to another hospital and underwent MCS and HT, patient #4 (chronic myocarditis) was treated successfully in our hospital with the intravenous infusion of inotropic medications. A similar clinical problem occurred in patient #3 (acute myocarditis), who presented a late acute deterioration of cardiac function, without any infectious cause, and required hospitalization with infusion of inotropic agents. On the contrary, patient #5 (acute myocarditis) has had an uneventful post-operative course and could be discharged to his country of origin.

We suggest that a very close clinical and therapeutic follow-up (weekly evaluation and TTE assessment) is necessary for the first months after PAB, to avoid acute left ventricle deterioration due to common pediatric problems (i.e., pneumonia). The reason for this is uncertain, but we speculate that it is due to the slow regeneration process of cardiomyocytes, which requires time for a satisfactory myocardial recovery.

It is of note that patient #2 did not benefit at all of the PAB, and despite changed intracardiac mechanics (showed by daily TTE monitoring), the clinical condition and cardiac function did not improve. Unlike the other patients, he was the only patient older than 1 year of life (3.5 years) and had an extremely compromised hemodynamic state on admission in our center (sedated and intubated after a cardiogenic shock). Also, he was the only one who presented with a very dilated left atrium compressing the main left stem bronchus and causing air entrapment and lung damage. Owing to this, he underwent PAB with associated atrial septectomy and ECMO support. Despite weaning from ECMO, there was no improvement in cardiac function, and he finally underwent VAD support and HT. Because the presence of a dilated left atrium may be the evidence of a long-lasting myocardial dysfunction before the onset of symptoms, we speculate that this particular patient might have acute myocarditis on an undetected idiopathic DCM. Thus, the long-lasting myocardial damage may have prevented a positive effect of PAB.

On the other hand, this failure might be due to a reduced regenerative myocardial capacity, which is known to correlate negatively with patients' age (33). Currently, two models describe that human infants and children generate new cardiomyocytes, with cardiomyocyte generation being highest in infancy, and declining to a relative stagnation in adults, with very low but measurable levels after 20 years of life (34–37). Even in adults, an adjustable PAB may be able to induce in selected patients a slower but effective improvement of sub-pulmonary left ventricle function, as described by our group in one 20-year-old lady with S/P Mustard in TGA (22).

On the basis of these data, we postulate that the potential for cardiomyocyte recovery and hyperplasia/repopulation may be the most significant in infancy, as elsewhere stated (34). Most patients in our study are <12 months of age. In the long term, PAB can reduce the ischemic-necrotizing injury of the left ventricle using a slow, gradual compensatory mechanism. Whether this process is due to vicariant viable myocyte hypertrophy or to circulating stem cells, myocardial repopulation is not guaranteed. However, it is widely known and accepted that in the first months of life, there are plenty of circulating multipotent stem cells in the blood circulation. Because the histological evaluation of human heart after successful PAB would be useful scientifically, but ethically contraindicated, we suggest to repeat MRI periodically to identify fibrotic areas or viable myocardium to evaluate myocardial disease recovery.

In our experience, the PAB procedure presents some crucial advantages. First of all, it is a simple surgical procedure, which does not require cardiopulmonary bypass, and can be tolerated hemodynamically by the patients as long as continuous echocardiographic monitoring is assured. Furthermore, there is the possibility to modulate PAB tightness both in the immediate post-operative period (by delayed chest closure) and by graded percutaneous balloon dilation of the band in the follow-up (when PAB is too narrow with high gradient trans-PAB and tricuspid regurgitation increases owing to right ventricle dilation). In fact, in our series, the last three patients underwent percutaneous balloon de-banding to preserve the right ventricle function. Third, when compared with VAD surgery, PAB is easier and less invasive. It does not require left ventricle apicectomy (for the VAD inflow cannula), which may jeopardize the left ventricle function recovery, as suggested by some studies focused on muscle layer contractility and the pivotal role of the left ventricle apex (38). Last, after successful PAB, the patient can be managed in the ward, mobilized, and discharged home with weekly hospital evaluations; and the infective and thromboembolic complications, which may occur during MCS, are minimized as well by PAB strategy.

**Figure 4:**
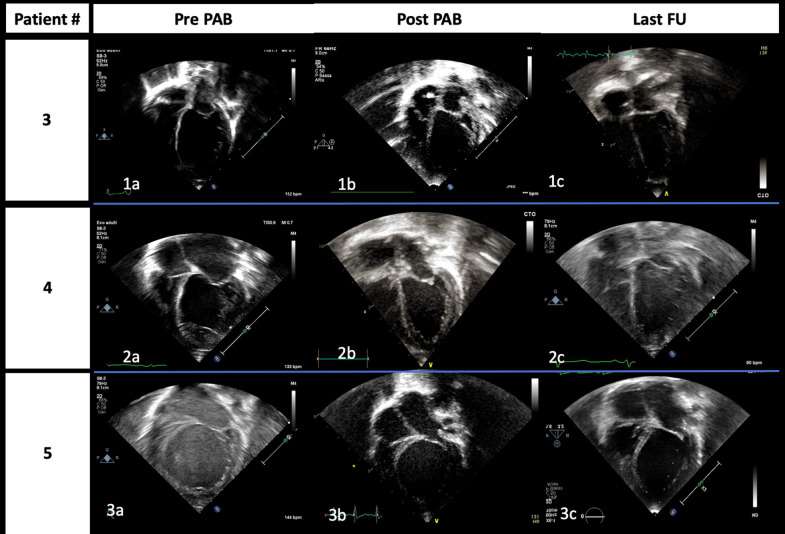
End-diastolic images from apical four-chamber view of patients #3 (1), #4 (2), and #5 (3) acquired at admission **(a)**, immediately after PAB procedure **(b)**, and at the last follow-up **(c)**, respectively. The gradual improvement of left atrium and ventricle dilation is highlighted; it is of note that it is already evident immediately after PAB.

In our experience, in four patients, the gradual clinical improvement and functional left ventricle recovery offered a chance for discharge home. Most importantly, three among five reached complete LVEF normalization (Figure 4), average somatic growth, and neuropsychological development out of the hospital, and they experienced such a dramatic progressive clinical improvement that allowed us to remove them from HT list.

Our study cannot clarify if PAB-induced cardiac remodeling was only a modality of transient support of the ventricular function in a framework of myocarditis that would have undergone a spontaneous resolution, or if it had a pathophysiological role (partial or more consistent) in the cardiomyopathy complete recovery. However, it certainly avoided that four among five patients would undergo a more invasive approach in the acute phase (such as MCS or, in extreme cases, emergent HT).

We conclude that PAB strategy has provided these children a possibility of complete remission without damaging irreversibly the cardiac structures or great vessels and an option to have a healthy life expectancy after recovery, without the drawbacks of life-lasting immunosuppressive therapy.

### Limitations

The significant limitations are the small number of patients and the short follow-up time, which prevents long-term cardiac function assessment.

## Conclusions

The introduction of PAB may represent a paradigmatic change in managing pediatric ESHF. In our experience, PAB resulted in an effective and affordable procedure with acceptable risk and convincing medium-term outcomes. We believe this strategy can be a valid option in selected infants and children with ESHF, as an alternative strategy to MCS for bridging to transplant or, hopefully, cardiac recovery. In our series, better results occurred in patients aged <12 months, probably owing to a preserved myocardial regenerative potential. However, the early post-operative period can be extremely challenging, and serious complications may be caused by transient diseases typical of infancy. Thus, the PAB strategy requires strict clinical follow-up and frequent hospital admissions and evaluations. Further experience and research are required to differentiate between “responder” and “non-responder” patients to this innovative heart failure therapy.

## Data Availability Statement

The datasets generated for this study are available on request to the corresponding author.

## Ethics Statement

The studies involving human participants were reviewed and approved by University hospital of Padua. Written informed consent to participate in this study was provided by the participants' legal guardian/next of kin.

## Author Contributions

All authors listed have made a substantial, direct and intellectual contribution to the work and approved it for publication.

## Conflict of Interest

The authors declare that the research was conducted in the absence of any commercial or financial relationships that could be construed as a potential conflict of interest.
